# Health‐Literate Healthcare Organization Attributes and Perceived Workload Among Nurses and Nursing Coordinators

**DOI:** 10.1155/jonm/3119702

**Published:** 2026-08-25

**Authors:** Valentina Simonetti, Giulia Franceschini, Chiara Lorini, Guglielmo Bonaccorsi, Gloria D’Angelo, Sonia Stante, Claudia Mezzadri, Martina Tesei, Giancarlo Cicolini, Dania Comparcini

**Affiliations:** ^1^ Department of Innovative Technologies in Medicine & Dentistry, “G. D’Annunzio” University of Chieti-Pescara, Chieti, Italy, unich.it; ^2^ Administrative Healthcare Databases and Monitoring of Regional Healthcare System Sector, Regional Health Agency of Marche Region, Ancona, Italy; ^3^ Department of Health Science, University of Florence, Florence, Italy, unifi.it; ^4^ Faculty of Medicine and Surgery, Università Politecnica Delle Marche, Ascoli Piceno, Italy, univpm.it; ^5^ Emergency Department, Local Health Authority of Ancona, Senigallia, Italy; ^6^ Primary Health Care, Local Health Authority of Macerata, Civitanova Marche, Italy; ^7^ Interdisciplinary Department of Medicine, Aldo Moro University of Bari, Bari, Italy, uniba.it

**Keywords:** cross-sectional, organizational health literacy, quality of care, workload

## Abstract

**Background:**

Health‐literate healthcare organizations (HLHOs) have been associated with improved clinical outcomes and quality of care for both patients and healthcare services, through the implementation of the “Ten Attributes of HLHOs” which support individuals in navigating, understanding, and using health information and services. Nurses, in both clinical and managerial roles, are crucial in supporting HLHO initiatives. However, a high level of perceived workload may influence their engagement with health literacy–related initiatives.

**Purpose:**

To assess nurses’ and nursing coordinators’ perceptions of the implementation of measures aimed at achieving HLHO status and the relationship between HLHO, sociodemographic factors, and participants’ perceived workload.

**Methods:**

A multicenter, cross‐sectional study was conducted among nurses and nursing coordinators employed in four Italian facilities. An online questionnaire was used to collect participants’ sociodemographic data, perceived workload, and perceptions of the implementation of the ten HLHO attributes using the HLHO‐10 instrument.

**Results:**

A total of 386 participants were included. The HLHO‐10 final scores revealed moderate perceptions of the implementation of health literacy measures across participating centers (mean = 4.73 out of 7; SD = 1.93). Higher HLHO levels were significantly associated with lower perceptions of workload. Female nurses and nurses employed in critical care and emergency departments were less likely to report lower workload (all *p* < 0.001).

**Conclusions:**

Higher HLHO levels were associated with lower perceived workload, while differences were observed according to gender and working area. These findings suggest that organizational and contextual factors may be related to workload perceptions and highlight areas for further investigation and potential organizational improvement.

**Implications for Nursing Management:**

Healthcare organizations should consider integrating HLHO principles into organizational policies and practices while supporting staff education and organizational development. Stronger implementation of HLHO principles may be associated with more favorable workload perceptions and staff well‐being. Nurse managers and nursing coordinators can play an important role in promoting OHL initiatives and fostering organizational environments that support both healthcare professionals and high‐quality patient care.

## 1. Introduction

Safety and quality of care are widely recognized as priority objectives for healthcare systems worldwide, as they represent fundamental pillars of the right to health for both individuals and communities [1]. Efforts to improve organizational conditions and address system‐level factors may support patients and healthcare consumers in making informed health decisions, potentially reducing the risk of medical errors, inappropriate healthcare utilization, and adverse health outcomes [2].

In this scenario, a work environment characterized by supportive organizational conditions has been associated with more favorable employee outcomes, which may in turn contribute to positive organizational and patient‐related outcomes [3].

Promoting such environments requires attention to the informational dimension of structural empowerment. This dimension refers to the organizational structure that enables healthcare professionals (HCPs) to access relevant information, critical data, technical knowledge, and skills necessary to perform their roles effectively and achieve professional goals. Access to these resources may support informed decision‐making, professional autonomy, and effective clinical practice [4].

In this context, organizational health literacy (OHL), which refers to the extent to which healthcare services, organizations, and systems enable individuals to access, understand, appraise, and use health‐related information and services, while also supporting HCPs through clear communication and accessible information resources [5], may provide a useful framework for reflecting on the promotion of workplace empowerment.

OHL seeks to enhance the accessibility, usability, and clarity of information within healthcare organizations, factors that have been associated with improvements in the quality of care [6]. By facilitating access to relevant information and promoting effective communication processes, OHL may contribute to organizational environments in which HCPs feel better supported in managing job demands. In turn, such environments may foster professional autonomy, informed decision‐making, and greater engagement in clinical and organizational activities, all of which are key components of workplace empowerment.

OHL plays a pivotal role in addressing challenges related to individual health literacy (IHL) through adequate communication and educational activities tailored to the needs of patients and communities [7]. Evidence suggests that health‐literate healthcare organizations (HLHOs) are associated with higher patient satisfaction, greater patient empowerment, enhanced decision‐making capabilities, and a more active role in treatment processes [8]. Furthermore, HLHOs have been linked to improvements in overall quality of healthcare [9, 10] and to favorable economic and health‐related outcomes, including disease prevention, lower hospitalization rates and associated costs [11], increased participation in recommended health screenings [12], more appropriate use of healthcare services [13], and efforts to reduce sociohealth inequalities [14].

The implementation of OHL within healthcare organizations is a complex and dynamic process, rather than a simple adoption of predefined standards [15]. Evidence from implementation science suggests that this process is influenced by factors such as organizational readiness, leadership commitment, resource availability, and other contextual characteristics that affect the integration of OHL into routine care [16]. Consequently, the extent and nature of OHL implementation may vary across healthcare settings according to organizational maturity and the degree to which HL principles are embedded within governance structures, organizational policies, and clinical processes [15].

For healthcare organizations, the key characteristics of becoming an HLHO, namely, facilitating individuals’ ability to navigate, understand, and use information and services to take care of their health, are summarized in the “Ten Attributes of Health Literate Health Care Organizations” [2]. This framework outlines organizational components intended to address the challenges associated with limited health literacy and support the accessibility, quality, safety, and value of healthcare services. Nurses, who constitute the largest professional group within the healthcare workforce, play a central role in promoting health literacy and supporting the implementation of HLHO principles in both clinical and managerial roles [7]. Through patient communication, education, advocacy, and caring relationships, nurses contribute substantially to health literacy‐related initiatives and to the delivery of patient‐centered care [7, 17].

The implementation of OHL is increasingly supported by institutional efforts aimed at strengthening health communication and information systems. However, healthcare organizations show substantial variability in the maturity and integration of OHL‐related strategies and practices [6]. Such variability may influence how HCPs perceive and experience OHL attributes within their organizations. Evidence suggests that these perceptions are influenced by the organizational context, particularly by the extent to which OHL initiatives are implemented, integrated, and sustained in routine practice [6, 7, 15].

However, previous studies have reported that nurses working under high workload conditions, particularly in resource‐constrained environments, may perceive their organization’s commitment to HL as less effective [18] or insufficient [19]. Under these circumstances, nurses may prioritize clinical and task‐oriented activities over communication and relational aspects of care, such as patient–provider interactions, which are central components of HL initiatives [7, 20].

Several recent studies, conducted in both non‐European [18, 21–23] and European contexts [24–27], have explored HCPs’ perceptions of OHL. However, to our knowledge, most of these studies have not specifically focused on nurses as a distinct professional group [21, 22, 24–26, 28, 29] and have frequently been conducted in inpatient settings only [21, 22, 24–26]. Furthermore, although OHL perceptions have been investigated among HCPs across a variety of healthcare contexts, including primary healthcare settings [19, 23, 27–29], nurses’ perceptions of the attributes of OHL have received limited attention. In addition, the perspectives of nursing coordinators, who play a key role in organizational leadership and in the implementation of health literacy‐related practices, remain largely unexplored.

To date, although OHL has been associated with healthcare quality and patient‐related outcomes [9, 10, 26], evidence regarding its relationship with perceived workload among nurses and nursing coordinators remains limited.

This study aimed to assess nurses’ and nursing coordinators’ perceptions of the implementation of measures intended to support HLHOs and to examine the association between these measures and perceived workload.

## 2. Materials and Methods

### 2.1. Study Design, Participants, and Setting

A multicenter, cross‐sectional study was conducted between May 2023 and January 2024 among Italian nurses and nursing coordinators across four public healthcare institutions in the Marche Region: IRCCS INRCA (National Institute of Health and Science on Aging) and the Aziende Sanitarie Territoriali (AST; Local Health Authorities) of Ancona, Ascoli Piceno, and Pesaro‐Urbino.

IRCCS INRCA (structure A) is a national research and healthcare institute specializing in geriatric care and aging‐related research. Its main hospital has approximately 170–180 inpatient beds and serves adults aged ≥ 65 years across the region (population served: 388.839) [30].

AST of Ascoli Piceno (structure B) delivers integrated hospital and territorial care through two acute‐care hospitals with approximately 500 accredited beds, covering a population of 200.897 inhabitants; AST of Pesaro‐Urbino (Structure C) manages a network of hospital and community services with approximately 880–890 inpatient beds, serving 349.882 residents; and AST of Ancona (Structure D) provides hospital and community services through multiple facilities, including acute‐care hospitals with approximately 750–760 inpatient beds, serving about 461.629 residents [30].

The ASTs provide comprehensive, multispecialty services, including emergency care, intensive care, oncology, and rehabilitation. In contrast, IRCCS INRCA offers specialized services focused on geriatric and chronic care, alongside selected medical specialties and rehabilitation.

This combination of organizations with different sizes, service profiles, and population catchment areas provided a heterogeneous context for evaluating OHL attributes.

Participants were recruited through convenience sampling based on the following inclusion criteria: (1) nurses with clinical or coordination roles and (2) provision of consent to participate in the study by completing and signing the informed consent form. The reporting of this observational study adheres to the STROBE Statement [31], which provides guidance for transparent reporting of observational research (Supporting File 1).

### 2.2. Sample Size

The reference population for the sample size calculation comprised 6.327 nurses, and nursing coordinators employed across the participating healthcare institutions. The sample size was calculated for the descriptive objective of the study, that is, to estimate the mean HLHO score with adequate precision. Based on a mean HLHO‐10 score of 5.37 (SD = 1.51), as reported by Rathmann et al. [26], and assuming a 99% confidence level with finite population correction, the minimum required sample size was estimated at 168 participants. The multivariable logistic regression examining the association between HLHO‐related measures and perceived workload was conducted as a secondary exploratory analysis. Since no previous studies provided reliable estimates of the expected association between HLHO scores and perceived workload, no a priori, regression‐based power calculation was performed.

### 2.3. Study Procedure, Sampling, and Data Collection

Data were collected through the electronic Webropol survey and Reporting [32]. A trained researcher supervised the data collection process. A nonprobability convenience sampling approach was adopted, inviting participants on a voluntary basis.

The survey link, along with an information sheet detailing the study aims, methods, and data protection procedures, was distributed to the directors of the participating healthcare institutions. Directors were asked to disseminate the invitation to all eligible nurses and nursing coordinators within their organizations. As the dissemination process was managed locally by each institution and the number of professionals who actually received and opened the invitation was not centrally monitored, the exact denominator of invited participants was unknown. Consequently, a response rate could not be reliably calculated.

To enhance participation, two reminder emails were sent at 2‐week intervals. The online questionnaire was protected by CAPTCHA verification and cookie‐based controls to minimize duplicate responses. Participation was voluntary, and informed consent was obtained electronically prior to survey completion.

### 2.4. Instruments

The questionnaire comprised two sections. The first collected sociodemographic and professional information and included a single‐item question on perceived workload assessed on a 3‐point Likert scale (1 = *low*, 2 = *medium*, 3 = *high*). The second section consisted of the validated Italian version [26] of the HLHO‐10 questionnaire [33], which assesses HCPs’ perceptions of the extent to which the ten attributes of an HLHO are implemented within their own workplace. Each item refers to a specific organizational attribute and asks respondents to rate the extent to which that attribute is realized in their facility, thus reflecting the perceived level of implementation. Responses are provided on a 7‐point Likert scale ranging from 1 (*completely disagree*) to 7 (*completely agree*), with an additional “not applicable” (NA) option. Higher scores reflect a more positive perception of the degree of implementation of HLHO attributes [34]. In this study, the Cronbach’s alpha of the HLHO‐10 was 0.91.

### 2.5. Ethical Considerations

The study received ethical approval from the Institutional Review Board of the Marche Region (protocol n*o*. 249/2023) and permission from the participating healthcare institutions.

### 2.6. Statistical Analysis

Data were presented as percentages, means with standard deviations (SD), or medians with interquartile ranges (IQR).

Associations between workload levels and other variables collected were assessed using the chi‐square test or ANOVA, as appropriate.

Multivariable logistic regression analysis was performed to evaluate the extent to which HLHO‐10 scores and other variables collected influenced workload. This analysis was intended as a secondary and exploratory analysis. Workload levels (the dependent variable) were dichotomized, and the likelihood of low versus medium/high workload was calculated as an odds ratio (OR). Variables that showed a significant association with workload in the bivariate analysis were included as covariates (independent variables).

Two multivariable logistic regression models were tested. In Model 1, each individual HLHO‐10 item was included; in Model 2, the overall HLHO‐10 score was used instead of the individual items.

For all analyses, a significance level (alpha) of 0.05 was applied. Analyses were conducted using IBM SPSS Statistics Version 29.0. Collinearity diagnostics were examined for the predictors included in the item‐level model. Variance inflation factors (VIFs) and tolerance values were estimated using an auxiliary linear regression model including the same independent variables as the logistic regression model. This approach was used because the VIF reflects the degree of linear association among predictors, independently of the distribution of the outcome variable.

## 3. Results

### 3.1. Sample Characteristics

A total of 386 participants completed the questionnaire. Nurses constituted the largest professional group among respondents (86.1%), and nearly half of the sample (47.9%) reported more than 20 years of professional experience. Overall, 26.7% of participants reported a low workload, 48.7% reported a medium workload, and 24.6% reported a high workload; 13.9% were coordinators (Supporting File 2).

### 3.2. Descriptive Analysis

The mean HLHO‐10 score was 4.73 (*SD* = 1.93), and the mean scores of the HLHO‐10 items ranged from 3.90 to 5.19 on a 7‐point scale (Table 1). Coordinators reported significantly higher scores than noncoordinators for the HLHO3 item (“*Is health information at your hospital developed by involving patients?*”), the HLHO4 item (“*Is individualized health information used at your hospital, e.g., different languages, print sizes, Braille?*”), and the overall HLHO‐10 score. Specifically, the mean HLHO3 score was 5.30 (SD = 2.05) among coordinators and 4.56 (SD = 2.23) among noncoordinators (*p* = 0.027). The mean HLHO4 score was 5.74 (SD = 1.67) among coordinators and 5.04 (SD = 2.08) among noncoordinators (*p* = 0.019). Finally, the mean HLHO‐10 score was 5.30 (SD = 1.61) among coordinators and 4.64 (SD = 1.97) among noncoordinators (*p* = 0.020).

**TABLE 1:** Scores at the HLHO‐10: descriptive analysis and items by workload level.

HLHO‐10 items	HLHO‐10		Workload level				
			Low (*N* = 103)	Medium (*N* = 188)	High (*N* = 95)	*p* value^∗^	*F*
	Mean (SD)	Median (IQR)	Mean (SD)	Mean (SD)	Mean (SD)		
HLHO1: Is the management at your hospital explicitly dedicated to the subject of health literacy (e.g., mission statement, human resources planning)?	4.48 (2.19)	5 (2–75−6)	5.06 (1.98)	4.38 (2.22)	4.04 (2.24)	0.003	5.817
HLHO2: Is the topic of health literacy considered in quality management measures at your hospital?	4.31 (2.25)	4 (2–6)	5.00 (2.20)	4.09 (2.26)	3.99 (2.17)	0.001	6.882
HLHO3: Is health information at your hospital developed by involving patients?	4.66 (2.22)	5 (2–7)	5.45 (2.08)	4.34 (2.16)	4.44 (2.29)	< 0.001	9.276
HLHO4: Is individualized health information used at your hospital (e.g., different languages, print sizes, braille)?	5.13 (2.04)	6 (4–7)	5.73 (1.68)	4.93 (2.08)	4.89 (2.21)	0.003	6.086
HLHO5: Are there communication standards at your hospital which ensure that patients truly understand the necessary information (e.g., translators, allowing pauses for reflection, calling for further queries)?	4.77 (2.19)	5 (3–7)	5.46 (1.88)	4.58 (2.27)	4.40 (2.19)	< 0.001	7.342
HLHO6: Are efforts made to ensure that patients can find their way at your hospital without any problems (e.g., direction signs, information staff)?	4.87 (2.19)	6 (3–7)	5.62 (1.86)	4.77 (2.21)	4.25 (2.28)	< 0.001	10.537
HLHO7: Is information made available to different patients via different media at your hospital (e.g., three‐dimensional models, DVDs, picture stories)?	4.47 (2.06)	5 (2–6)	5.17 (1.78)	4.31 (2.15)	4.00 (2.00)	< 0.001	9.398
HLHO8: Is it ensured that the patients have truly understood everything, particularly in critical situations (e.g., medication, surgical consent), at your hospital?	5.04 (2.09)	6 (3–7)	5.60 (1.79)	4.87 (2.20)	4.76 (2.09)	0.005	5.325
HLHO9: Do you communicate openly and comprehensibly at your hospital to your patients in advance about the costs which they themselves have to pay for treatment (e.g., out‐of‐pocket payments)?	5.19 (2.19)	6 (4–7)	5.89 (1.81)	5.06 (2.26)	4.71 (2.26)	< 0.001	8.258
HLHO10: Are employees at your hospital trained on the topic of health literacy?	3.90 (2.33)	4 (2–6)	4.46 (2.39)	3.70 (2.33)	3.67 (2.20)	0.017	4.124
HLHO‐10 final score	4.73 (1.93)	5 (3–6)	5.49 (1.63)	4.52 (1.98)	4.33 (1.94)	< 0.001	11.883

Abbreviations: IQR = interquartile range, SD = standard deviation.

^∗^ANOVA.

### 3.3. Characteristics of the Study Population, HLHO‐10 Scores, and Perceived Workload

The HLHO‐10 final score varied significantly across workload categories (*p* < 0.001), with higher scores correlating with lower perceived workload (Table 1).

Bivariate analyses revealed significant associations between perceived workload levels and both gender and working area. The distribution of workload differed significantly by gender (*χ* ^2^(2) = 14.129, *p* < 0.001). In addition, workload levels varied significantly across different working areas (*χ* ^2^(14) = 45.238, *p* < 0.001) (Supporting File 3). Notably, no significant association was found between perceived workload and the “coordinator” variable.

### 3.4. Impact of OHL on Workload

Table 2 shows two multivariable logistic regression models examining the impact of HLHO‐10 items and the overall final score on perceived workload (low vs. medium/high), adjusting for gender and working area.

**TABLE 2:** Multivariate logistic regression analysis.

Variables	Model 1			Model 2		
	OR	(95% CI)	*p* value	OR	(95% CI)	*p* value
*Gender*						
Male	Ref.	—	—	Ref.	—	—
Female	**0.41**	**(0.25; 0.68)**	**< 0.001**	**0.44**	**(0.27; 0.72)**	**0.001**

*Working area*						
Critical/emergency	Ref.	—	—	Ref.	—	—
Medical	1.80	(0.73; 4.45)	0.199	1.75	(0.72; 4.24)	0.215
Surgical	2.33	(0.85; 6.36)	0.100	2.27	(0.85; 6.07)	0.103
Geriatric	**3.56**	**(1.39; 9.14)**	**0.008**	**3.42**	**(1.38; 8.47)**	**0.008**
Community	**3.89**	**(1.39; 10.91)**	**0.010**	**3.67**	**(1.35; 9.96)**	**0.011**
Maternal and child	**10.21**	**(2.69; 38.79)**	**< 0.001**	**9.65**	**(2.60; 35.77)**	**< 0.001**
Mental health	1.26	(0.36; 4.44)	0.722	1.24	(0.36; 4.22)	0.730
Outpatient	**4.40**	**(1.71; 11.38)**	**0.002**	**4.27**	**(1.69; 10.76)**	**0.002**

*HLHO-10 items*						
HLHO1	0.99	(0.82; 1.20)	0.919	—		—
HLHO2	1.05	(0.86; 1.28)	0.628	—		—
HLHO3	**1.21**	**(1.00; 1.45)**	**0.048**	—		—
HLHO4	1.00	(0.82; 1.21)	0.972	—		—
HLHO5	0.97	(0.81; 1.17)	0.758	—		—
HLHO6	1.08	(0.88; 1.31)	0.463	—		—
HLHO7	1.09	(0.90; 1.33)	0.367	—		—
HLHO8	0.95	(0.80; 1.13)	0.574	—	—	—
HLHO9	1.13	(0.98; 1.31)	0.092	—	—	—
HLHO10	0.95	(0.82; 1.09)	0.450	—	—	—
**HLHO-10 final score**	—	—	—	**1.35**	**(1.17; 1.57)**	**< 0.001**

*Note*: Dependent variable: workload (low vs. medium/high). Bold values indicate statistically significant values.

Abbreviations: CI = confidence interval, OR = odds ratio.

In Model 1 (each HLHO‐10 item included), female participants were more likely to report higher workload compared to males (OR = 0.41, 95% CI [0.25, 0.68], *p* < 0.001), suggesting potential differences in perceived work demands across sex.

Concerning the working area, compared to the reference category (critical/emergency area), participants in the maternal and child (OR = 10.21, 95% CI [2.69, 38.79], *p* < 0.001), outpatient (OR = 4.40, 95% CI [1.71, 11.38], *p* = 0.002), community (OR = 3.89, 95% CI [1.39, 10.91], *p* = 0.010), and geriatric settings (OR = 3.56, 95% CI [1.39, 9.14], *p* = 0.008) reported lower workload levels, which may reflect differences in care complexity or organizational structure compared to critical/emergency units. However, this estimate should be interpreted with caution because the maternal and child health subgroup included only 14 participants, resulting in a wide confidence interval and a potentially unstable odds ratio. No significant differences were observed for medical, surgical, and mental health settings.

Among the HLHO‐10 items, only item 3 “Is health information at your hospital developed by involving patients?” was significantly linked to workload. However, given the high internal consistency of the HLHO‐10 scale and the conceptual overlap among items, this item‐level finding should be interpreted cautiously. Collinearity diagnostics did not indicate severe multicollinearity among the HLHO‐10 items included in Model 1. VIF values ranged from 1.43 for item 9 to 3.23 for item 2 of the HLHO‐10, and tolerance values ranged from 0.310 for item 9 to 0.70 for item 2 of the HLHO‐10. Although these values suggest some shared variance among HLHO‐10 items, they remained below commonly used thresholds for severe multicollinearity. Nonetheless, Model 1 should be considered an exploratory item‐level model.

Model 2 (HLHO‐10 final score included) showed that a higher HLHO‐10 final score was significantly associated with increased likelihood of reporting low workload (OR = 1.35, 95% CI [1.17, 1.57], *p* < 0.001). Overall, the results for gender and working area were similar to those in Model 1.

## 4. Discussion

OHL constitutes a critical foundation for health‐promoting hospitals and has been proposed as an organizational approach to improving communication and reducing barriers to care. It involves restructuring care and services to support individuals in navigating and understanding health information more easily [7]. By addressing the needs of patients with diverse literacy skills, OHL may contribute to more effective navigation within complex healthcare systems. This aligns with hospitals’ goals of promoting patient education, empowerment, and accessible care environments, factors that have been associated with improved health outcomes [24].

This study advances the understanding of HLHO development, which is crucial for improving patient experience and satisfaction [23]. It offers insights into both OHL efforts and their association with professionals’ perceived workload.

Overall, the HLHO‐10 final scores revealed moderate perceptions of the implementation of health literacy measures across participating centers, in agreement with previous studies involving HCPs, including nurses [21, 25–27]. Items related to personalized patient information (e.g., use of multilanguage materials, HLHO4), communication standards for understanding (HLHO5), and implementation of efforts for patients to navigate and orient themselves within the hospital environment (HLHO6) scored relatively high, with the item HLHO4 showing the highest score. Additionally, item HLHO9, which assesses the ability to communicate openly and comprehensibly with patients about their out‐of‐pocket treatment costs, received one of the highest mean scores.

Interestingly, the results of this study are consistent with those of two studies from Germany [25, 27], but they differ from those of a recent study conducted outside Europe [21]. This probably mirrors the Italian Public Healthcare System, where inpatient and primary care are usually free, but copayments are required for certain specialist visits and medications [35]. This arrangement promotes transparent conversations between patients and providers regarding costs, a pattern also observed in Germany, which has a mixed public‐private healthcare system with statutory health insurance covering the majority of the population [35]. The interpretation of the findings should consider the organizational context of the participating hospitals [6, 7, 15], which represented a heterogeneous sample of healthcare settings with varying levels of organizational maturity and implementation of OHL initiatives. Consequently, perceptions of HLHO attributes may be influenced by both existing organizational structures and ongoing efforts to strengthen OHL. This (unmeasured) contextual variability may have affected respondents’ perceptions and partly explained differences in perceived implementation across organizations. These considerations are consistent with implementation science perspectives, highlighting the role of contextual factors in shaping the adoption and integration of organizational practices [15, 16].

The lowest scores, for employee HL training (HLHO10), reveal a notable gap, consistent with prior studies [22, 25, 26], indicating inadequate nurse training on HL despite organizational efforts. Häberle et al. [24] found that the academic hospital scored higher on Standard 8, assessing staff HL training, local hospitals in their Italian OHL study, which used the International Self‐Assessment Tool for OHL of Hospitals.

The higher HLHO‐10 final score, HLHO3, and HLHO4 scores reported by coordinators may indicate a greater perceived presence of OHL practices among professionals with managerial responsibilities. As coordinators are often involved in quality improvement activities and organizational decision‐making processes, they may be more familiar with initiatives aimed at engaging patients in the development of health information and tailoring information to diverse patient needs [2]. The higher scores reported by coordinators may reflect role‐related differences in organizational perspective. Previous research has shown that nurse managers and staff nurses often differ in their perceptions of leadership and implementation climate, with managers generally reporting more positive assessments of organizational processes [36].

Nevertheless, these findings should be interpreted with caution, as they may reflect differences in role‐specific knowledge and organizational perspective rather than objective differences in health literacy responsiveness.

Regarding sample characteristics and workloads, gender and working area were associated with perceived workload among nurses and coordinators: higher workload levels were more frequently reported by women and by staff working in acute care areas, suggesting that both sex and clinical setting may influence the perception of workload demands. Notably, no significant association was found between perceived workload and professional role, indicating that perceived workload was similar between nurses and coordinators.

Logistic regression confirms the results from the bivariate analysis: females are approximately half as likely as males to perceive a low workload, highlighting potential disadvantages in the workplace, particularly in terms of work–home balance, as reported in previous evidence [37]. Such challenges may influence their perception of workload and overall occupational well‐being, highlighting the need for workplace policies that address these gender‐specific issues.

Additionally, compared with the critical/emergency departments, nurses working in geriatric, community, outpatient, and maternal and child settings were more likely to report a low workload. This pattern suggests that the perception of workload varies across clinical contexts rather than being tied to a single setting. These differences may reflect variation in patient complexity, care intensity, and organizational structures across services. Overall, these findings suggest that structural and organizational characteristics of healthcare environments may be associated with nurses’ perceptions of workload. To date, the literature has not concurrently explored the association between specific healthcare settings and perceived workload, although studies have examined its relationship with OHL [22, 23]. Nonetheless, as healthcare settings differ in terms of patient volume and complexity of care, these factors are likely to influence nurses’ workload perceptions.

The item‐level analysis (Model 1) suggested a possible association between HLHO item 3, concerning the involvement of patients in the development of hospital health information, and perceived workload. However, this finding was marginally significant, was not corrected for multiple comparisons, and emerged from an exploratory model including highly related HLHO‐10 items. Therefore, it should be interpreted as hypothesis‐generating rather than confirmatory.

Additionally, Model 2 showed that when considering the total HLHO‐10 score as a comprehensive measure of OHL, higher HLHO‐10 scores were associated with a greater likelihood of reporting lower perceived workload. However, this association should be interpreted with caution. Due to the cross‐sectional design, the temporal relationship between variables cannot be established, which precludes conclusions regarding directionality or causality [38, 39]. It is possible that HCPs working in less strained and better‐resourced environments may have greater capacity to implement, recognize, and positively evaluate OHL‐related practices. In this scenario, lower perceived workload may facilitate the development and perception of OHL attributes rather than OHL implementation being associated with lower workload perceptions. Furthermore, both perceptions may be influenced by broader organizational factors, such as leadership, staffing adequacy, and workplace culture, which are known to shape nurses’ work experiences and workload perceptions [40, 41]. Therefore, the observed association may reflect shared underlying organizational characteristics rather than a direct relationship between OHL and perceived workload.

These findings are particularly important, considering recent evidence suggesting that increasing workload negatively impacts the quality of care, job satisfaction, and symptoms of burnout [42]. Moreover, stronger implementation of OHL principles in clinical environments may be associated with organizational conditions linked to lower fatigue, which is widely recognized in the literature as a negative outcome associated with high workload. A recent study [43] found a strong correlation between perceived nursing workload and occupational fatigue, impacting physical and mental health, job satisfaction, and patient care quality.

The implementation of OHL principles is strongly influenced by organizational leadership and management capacity [7]. Effective leadership can facilitate the integration of HL‐oriented practices into routine care and may support communication, coordination, and workflow clarity [6]. Such organizational characteristics may also be associated with more efficient workforce organization and lower perceived workload among nurses. Therefore, leadership and management should be considered key contextual factors shaping both OHL implementation and workload perceptions.

These dynamics can also be interpreted through the lens of workplace empowerment. According to Kanter’s theory of structural empowerment, access to information, resources, and support within an organization enhances employees’ autonomy and effectiveness [44]. In this perspective, OHL can be conceptualized as part of the informational infrastructure that supports structural empowerment by ensuring access to clear and usable information within healthcare organizations [45]. Accordingly, higher levels of HLHO implementation may reflect more supportive organizational environments associated with greater autonomy and effectiveness among nurses, whereas lower levels may indicate less supportive contexts associated with higher levels of work strain.

Although not directly measured in this study, workplace empowerment may represent a potential mechanism linking OHL implementation to perceived workload. Future research should further explore these relationships by including key organizational mediators such as psychological and structural empowerment and job satisfaction, in order to better understand the complex factors influencing nurses’ workload perceptions.

A comprehensive approach that fosters a culture of OHL as a broader organizational construct may be associated with workplace conditions that support more favorable workload perceptions among nurses. However, OHL initiatives may also require additional time, training, and organizational resources during implementation. Therefore, the relationship between OHL and workload is likely to be complex and may vary according to organizational context, available resources, and the stage of implementation.

As a result, focusing solely on isolated improvements in individual HLHO attributes may be insufficient to influence workload perceptions, whereas a more integrated organizational approach may be more relevant.

These findings highlight the importance for healthcare managers and leaders recognizing and supporting the implementation of OHL principles through organizational, regional, and national strategies [24]. Previous research has suggested that OHL is associated with improved clinical outcomes, greater patient satisfaction, and more positive experiences among HCPs [2, 6].

These considerations further emphasize the importance of the institutionalization of OHL within healthcare organizations. This process extends beyond the implementation of individual interventions and reflects the extent to which HL principles are embedded in organizational culture, policies, and daily clinical practice [6, 45]. From this perspective, OHL may be associated not only with positive patient outcomes and care quality, but also with favorable experiences among HCPs, particularly regarding job satisfaction, work organization, and perceived well‐being.

### 4.1. Strengths and Limitations

The study provides a comprehensive overview of OHL within healthcare settings and explores the relationship between HLHO attributes and nurses’ and coordinators’ perceived workload. However, several limitations need to be acknowledged when interpreting the findings: (1) the cross‐sectional design of the study precludes any conclusions regarding the direction or causality of the observed associations. While higher HLHO scores were associated with lower perceived workload, it cannot be determined whether stronger OHL implementation contributes to lower workload perceptions or whether professionals experiencing lower workloads are more likely to perceive OHL initiatives positively. Furthermore, unmeasured organizational and contextual factors may have influenced both HLHO perceptions and workload assessments; (2) since both the predictor (perceived OHL) and the outcome (perception of workload) were measured using a self‐administered questionnaire, the results may have been influenced by social desirability bias, despite the anonymity of the questionnaires. Furthermore, because both variables were assessed simultaneously by the same respondents using the same data collection method, the study is susceptible to common‐method bias. Respondents experiencing negative affect or dissatisfaction may have been more likely to evaluate multiple aspects of their work environment unfavorably, potentially strengthening the observed associations between OHL and perceived workload. Therefore, the reported relationships should be interpreted with caution, as they may partly reflect shared method variance rather than solely the underlying construct of interest; (3) perceived workload was assessed using a nonvalidated, single‐item self‐report measure, which may not fully capture the multidimensional nature of workload. Furthermore, for the purpose of statistical analysis, the workload measure was dichotomized, introducing an arbitrary threshold and potential loss of information. This approach may have reduced the variability captured by the original responses and limited the sensitivity of the analysis. In addition, the use of a single‐item, nonstandardized measure may also have reduced the reliability and accuracy of workload assessment, increasing the risk of measurement error. Consequently, the observed associations should be interpreted with caution. Future studies should employ validated multidimensional workload instruments to provide a more comprehensive and robust assessment of nurses’ perceived workload; (4) only public healthcare institutions were included; therefore, it remains unclear whether similar findings would be observed in other healthcare contexts, such as teaching/university hospitals or private and accredited healthcare facilities; (5) the study sample was recruited through a nonprobability convenience sampling approach. As the exact number of HCPs who received the survey invitation was not available, a response rate could not be calculated. These factors, together with the voluntary nature of participation, may have introduced selection bias, as individuals with the greatest interest in OHL or more favorable perceptions of their work environment may have been more likely to participate. Consequently, the generalizability of the findings to other healthcare settings and professional populations should be considered with caution. Future studies employing probabilistic sampling methods and including a broader range of healthcare organizations would help strengthen the external validity of the results.

An additional consideration is the potential influence of unmeasured organizational and workforce‐related factors. Differences observed across clinical settings, particularly between critical/emergency departments and other care areas, may reflect variation in staffing adequacy, shift organization, professional experience, organizational size, leadership practices, and resource availability. Because these variables were not collected in the present study, they could not be included in the multivariable analyses. As a result, residual confounding cannot be excluded, and the observed associations between HLHO measures and perceived workload may partly reflect broader organizational characteristics rather than OHL‐related factors alone. Future research should incorporate these variables to better understand the independent contribution of OHL to HCPs’ workload perceptions.

Moreover, this study was primarily designed and powered to estimate nurses’ and nursing coordinators’ perceptions of HLHO‐related measures with adequate precision. The regression analysis examining the association between HLHO‐related measures and perceived workload was secondary and exploratory in nature. Consequently, the findings of the multivariable model, particularly those involving small subgroups, should be interpreted with caution and considered hypothesis‐generating rather than confirmatory.

Finally, some estimates related to working areas should be interpreted cautiously because of sparse data in certain categories. In particular, the maternal and child health subgroup included a limited number of participants, which may have resulted in sparse data bias and unstable odds ratio estimates. As a result, findings specific to this subgroup should be regarded as exploratory and require confirmation in larger studies with more balanced distributions across clinical settings.

Despite these limitations, the use of a validated instrument to measure HLHO is helpful for comparing results across different countries and settings, and for identifying areas for intervention to enhance OHL.

### 4.2. Linking Evidence to Action

- Nurses and nursing coordinators reported a moderate perception of the implementation level of the ten HLHO attributes within their healthcare organizations.
- Gaps in staff training on HL indicate a need for ongoing educational initiatives to support the development and maintenance of OHL.
- Female nurses and professionals working in critical and emergency care settings were less likely to report lower perceived workload, suggesting that gender and clinical context may influence workload perception.
- Healthcare organizations and policymakers should consider integrating HLHO principles into organizational strategies, as stronger OHL implementation may be associated with more favorable workload perceptions and nurse well‐being.
- Developing comprehensive OHL strategies that address multiple HLHO attributes, strengthen leadership and nursing management capacities, and promote supportive organizational environments may contribute to workplace conditions associated with perceived workload and sustainable nursing practice.

### 4.3. Implications for Nursing Management

Nurse managers and nursing coordinators play a pivotal role in fostering OHL through staff training, clear communication systems, and the provision of accessible patient information. They should also monitor workload distribution and evaluate potential differences across gender and clinical areas. Greater integration of OHL principles may be associated with lower perceived workload among nurses and may support organizational conditions that promote nurse well‐being, quality of care, and positive patient outcomes.

## 5. Conclusions

This study found that Italian nurses and nursing coordinators in the Marche Region generally perceived their healthcare organizations’ implementation of the 10 HLHO attributes positively.

Overall, higher OHL levels were associated with lower perceptions of workload. Moreover, female nurses and nurses employed in critical and emergency departments were less likely to report lower workload. The observed association between HLHO implementation and lower perceived workload suggests that organizations with stronger OHL characteristics provide organizational conditions associated with more favorable workload perceptions.

These findings highlight the potential value of integrating OHL principles into healthcare organizations, as such principles may support work environments associated with lower perceived workload among nurses. Healthcare providers may, therefore, consider organizational strategies that promote OHL principles as a means of fostering workplace conditions associated with nurse well‐being and high‐quality patient care. However, given the cross‐sectional nature of this study, no causal inferences can be drawn, and further longitudinal and interventional studies are needed to better understand the direction and nature of the relationship between OHL implementation and perceived workload.

## Author Contributions

Conceptualization: Giulia Franceschini, Valentina Simonetti, and Dania Comparcini; methodology: Giulia Franceschini, Valentina Simonetti, and Dania Comparcini; formal analysis: Chiara Lorini; investigation: Giulia Franceschini, Gloria D’Angelo, Sonia Stante, Claudia Mezzadri, and Martina Tesei; data curation: Chiara Lorini, Giulia Franceschini, Valentina Simonetti, and Dania Comparcini; writing–original draft: Valentina Simonetti, Dania Comparcini, and Giulia Franceschini; writing–review and editing: all authors; supervision: Guglielmo Bonaccorsi and Giancarlo Cicolini.

## Funding

This research received no specific grant from any funding agency in the public, commercial, or not‐for‐profit sectors. Open access publishing was facilitated by the Università degli Studi Gabriele d’Annunzio Chieti Pescara, as part of the Wiley‐CRUI‐CARE agreement.

## Disclosure

All authors have read and approved the final manuscript.

## Conflicts of Interest

The authors declare no conflicts of interest.

## Supporting Information

Additional supporting information can be found online in the Supporting Information section.

## Supporting information


**Supporting Information** The present Supporting Information is intended to complement the main manuscript and provide extended data, analyses, and methodological clarifications: Supporting file 1: STROBE Statement: checklist for reporting of observational research. Supporting file 2: Table of characteristics of the study population. Supporting file 3: Table of the study population characteristics significantly associated with workload level in the bivariate analysis (namely, gender and working area).

## Data Availability

The data that support the findings of this study are available from the corresponding author upon reasonable request. The data are not publicly available because they contain information that could compromise the privacy of research participants.
