# Supplementary material for: Health‐Literate Healthcare Organization Attributes and Perceived Workload Among Nurses and Nursing Coordinators
Source: J Nurs Manag. 2026 Aug 25;2026:3119702. doi: 10.1155/jonm/3119702 (PMC13507702; doi:10.1155/jonm/3119702)
Supplement: Supplementary file 1 [file JONM-2026-3119702-s001.docx]

**Supplementary files.**

**Supplementary file 1.**

***STROBE Statement—Checklist of items that should be included in reports of cross-sectional studies***

|  | Item No | Recommendation | Page n. |
| --- | --- | --- | --- |
| **Title and abstract** | 1 | (*a*) Indicate the study’s design with a commonly used term in the title or the abstract | 1 |
|  |  | (*b*) Provide in the abstract an informative and balanced summary of what was done and what was found | 2-3 |
| Introduction | | |  |
| Background/rationale | 2 | Explain the scientific background and rationale for the investigation being reported | 4-6 |
| Objectives | 3 | State specific objectives, including any prespecified hypotheses | 6 |
| Methods | | |  |
| Study design | 4 | Present key elements of study design early in the paper | 6 |
| Setting | 5 | Describe the setting, locations, and relevant dates, including periods of recruitment, exposure, follow-up, and data collection | 6-7 |
| Participants | 6 | (*a*) Give the eligibility criteria, and the sources and methods of selection of participants | 6-8 |
| Variables | 7 | Clearly define all outcomes, exposures, predictors, potential confounders, and effect modifiers. Give diagnostic criteria, if applicable | N/A |
| Data sources/ measurement | 8* | For each variable of interest, give sources of data and details of methods of assessment (measurement). Describe comparability of assessment methods if there is more than one group | 8-9 |
| Bias | 9 | Describe any efforts to address potential sources of bias | 9 |
| Study size | 10 | Explain how the study size was arrived at | 7-8 |
| Quantitative variables | 11 | Explain how quantitative variables were handled in the analyses. If applicable, describe which groupings were chosen and why | 7-8 |
| Statistical methods | 12 | (*a*) Describe all statistical methods, including those used to control for confounding | 9 |
|  |  | (*b*) Describe any methods used to examine subgroups and interactions | 10 |
|  |  | (*c*) Explain how missing data were addressed | N/A |
|  |  | (*d*) If applicable, describe analytical methods taking account of sampling strategy | N/A |
|  |  | (*e*) Describe any sensitivity analyses | N/A |
| Results | | |  |
| Participants | 13* | (a) Report numbers of individuals at each stage of study—eg numbers potentially eligible, examined for eligibility, confirmed eligible, included in the study, completing follow-up, and analysed | 7-8 |
|  |  | (b) Give reasons for non-participation at each stage | N/A |
|  |  | (c) Consider use of a flow diagram | N/A |
| Descriptive data | 14* | (a) Give characteristics of study participants (eg demographic, clinical, social) and information on exposures and potential confounders | 9-10 |
|  |  | (b) Indicate number of participants with missing data for each variable of interest | N/A |
| Outcome data | 15* | Report numbers of outcome events or summary measures | 9-10 |
| Main results | 16 | (*a*) Give unadjusted estimates and, if applicable, confounder-adjusted estimates and their precision (eg, 95% confidence interval). Make clear which confounders were adjusted for and why they were included | TABLE 1  TABLE 2 |
|  |  | (*b*) Report category boundaries when continuous variables were categorized | N/A |
|  |  | (*c*) If relevant, consider translating estimates of relative risk into absolute risk for a meaningful time period | N/A |
| Other analyses | 17 | Report other analyses done—eg analyses of subgroups and interactions, and sensitivity analyses | N/A |
| Discussion | | |  |
| Key results | 18 | Summarise key results with reference to study objectives | 10-12 |
| Limitations | 19 | Discuss limitations of the study, taking into account sources of potential bias or imprecision. Discuss both direction and magnitude of any potential bias | 17-18 |
| Interpretation | 20 | Give a cautious overall interpretation of results considering objectives, limitations, multiplicity of analyses, results from similar studies, and other relevant evidence | 12-16 |
| Generalisability | 21 | Discuss the generalisability (external validity) of the study results | 16-17 |
| Other information | | |  |
| Funding | 22 | Give the source of funding and the role of the funders for the present study and, if applicable, for the original study on which the present article is based | N/A |

*Give information separately for exposed and unexposed groups.

| **Supplementary file 2.**  *Characteristics of the study population (N = 386).* | | |
| --- | --- | --- |
| ***Socio-demographic variables*** | ***n*** | ***%*** |
| **Age (years)** |  |  |
| < 30 | 59 | 15.3 |
| 31–45 | 161 | 41.7 |
| 46–60 | 134 | 34.7 |
| >60 | 32 | 8.3 |
| *Mean (SD)* | *44.42 (11.74)* | |
| **Gender** |  |  |
| Male | 165 | 42.7 |
| Female | 221 | 57.3 |
| **Educational level** |  |  |
| Undergraduate diploma | 135 | 35 |
| Bachelor’s degree | 191 | 49.5 |
| Master’s degree | 60 | 15.5 |
| **Professional experience (years)** |  |  |
| 0–10 | 103 | 26.7 |
| 11–20 | 98 | 25.4 |
| > 20 | 185 | 47.9 |
| ***Variables related to the professional dimension*** | ***n*** | ***%*** |
| **Healthcare institution*** |  |  |
| Structure A | 78 | 20.2 |
| Structure B | 95 | 24.6 |
| Structure C | 92 | 23.8 |
| Structure D | 121 | 31.4 |
| **Professional role** |  |  |
| Nurse | 332 | 86.1 |
| Nursing coordinator | 54 | 13.9 |
| **Working area** |  |  |
| Medical | 84 | 21.8 |
| Surgical | 53 | 13.7 |
| Critical/Emergency | 76 | 19.7 |
| Geriatric | 52 | 13.5 |
| Community | 37 | 9.6 |
| Maternal and Child | 14 | 3.6 |
| Mental health | 24 | 6.2 |
| Outpatient | 46 | 11.9 |
| **Perceived workload** |  |  |
| Low | 103 | 26.7 |
| Medium | 188 | 48.7 |
| High | 95 | 24.6 |
|  | | |

| **Supplementary file 3.**  *Characteristics of the study population significantly associated with workload level al bivariate analysis.* | | | | |
| --- | --- | --- | --- | --- |
| **Variables** | **Workload** | | | |
|  | **Low (N=104)** | **Medium (N=188)** | **High (N=95)** | **χ² (df);**  ***p-value*** |
|  | ***n (%)*** | ***n (%)*** | ***n (%)*** |  |
| **Gender** |  |  |  |  |
| Male | 60 (36.4) | 72 (43.6) | 33 (20.0) | 14.129 (2); p < .001 |
| Female | 43 (19.5) | 116 (52.5) | 62 (28.1) |  |
|  |  |  |  |  |
| **Working area** |  |  |  |  |
| Medical | 17 (20.2) | 46 (54.8) | 21 (25.0) | 45.238 (14); p < .001 |
| Surgical | 11 (20.8) | 28 (52.8) | 14 (26.4) |  |
| Critical/Emergency | 10 (13.2) | 34 (44.7) | 32 (42.1) |  |
| Geriatric | 19 (36.5) | 19 (36.5) | 14 (26.9) |  |
| Community | 13 (35.1) | 19 (51.4) | 5 (13.5) |  |
| Maternal and Child | 9 (64.3) | 5 (35.7) | 0 |  |
| Mental health area | 5 (20.8) | 16 (66.7) | 3 (12.5) |  |
| Outpatient | 19 (41.3) | 21 (45.7) | 6 (13.0) |  |
| *Note.* df: degree of freedom. | | | | |
